# Primary Needle-Knife Sphincterotomy for Biliary Access in Patients at High Risk of Post-Endoscopic Retrograde Cholangiopancreatography Pancreatitis

**DOI:** 10.1155/2021/6662000

**Published:** 2021-05-18

**Authors:** Jin-Seok Park, Seok Jeong, Don Haeng Lee

**Affiliations:** ^1^Division of Gastroenterology, Department of Internal Medicine, Inha University Hospital, Inha University College of Medicine, Incheon, Republic of Korea; ^2^National Center of Efficacy Evaluation for the Development of Health Products Targeting Digestive Disorders (NCEED), Inha University Hospital, Incheon, Republic of Korea; ^3^Department of Internal Medicine, Inha University Hospital, 27 Inhang-ro, Jung-gu, Incheon, 400-711, Republic of Korea; ^4^Utah-Inha DDS & Advanced Therapeutics Research Center, Incheon, Republic of Korea

## Abstract

**Methods:**

Forty patients with one or more risk factors for PEP were prospectively enrolled between June 2018 and November 2019. The cannulation was conducted in all patients using NKS as the primary cannulation technique. Success rate of biliary cannulation, biliary cannulation time, and adverse event rate were assessed.

**Results:**

Of the 40 patients enrolled, 34 patients underwent primary NKS after the screening. Nine patients had 1 risk factor for PEP, 7 had 2, 8 had 3, 7 had 4, and 3 had 5. The success rate of biliary access by NKS was 94.1% (32/34). The median procedure time for NKS and the total procedure time for stone removal or biliary drainage were 4.1 minutes (range, 0.5-25.2) and 11.3 minutes (range, 3.8–40.4), respectively. Adverse events occurred in two patients (minor bleeding, *n* = 1; hyperamylasemia, *n* = 1). No patient experienced PEP or perforation.

**Conclusion:**

NKS might be feasible as a primary cannulation procedure in patients at high risk of PEP. This trial is registered with KCT0004886 (03/06/2018).

## 1. Introduction

Endoscopic retrograde cholangiopancreatography (ERCP) has become a standard procedure for the diagnosis and treatment of pancreaticobiliary diseases [1]. Common bile duct (CBD) cannulation is mandatory for successful endobiliary treatment, and usually, transpapillary cannulation is the preferred method. However, post-ERCP pancreatitis (PEP) is the most common adverse event following the procedure with a range from 2% to 10% in nonselective cases and can cause substantial morbidity, mortality, and high medical costs [2]. Due to recent advances in endoscopic techniques, the successful biliary cannulation rate has been improved and the incidence of PEP has decreased. However, selective biliary cannulation using the standard ERCP technique reportedly fails in 5-15% of cases, even in experienced hands [3, 4]. Available options to improve the success rate include precut sphincterotomy techniques [5], double-guidewire technique [6], transpancreatic septotomy [7], physician-controlled guidewires [8], or endoscopic ultrasound-guided interventions like the rendezvous procedure [9] or hepatogastrostomy [10].

Needle-knife sphincterotomy (NKS) involves making an incision at the suprapapillary region extending to the ampulla of Vater orifice and was first introduced by Siegel in 1980 [11]. Several studies have shown that this technique significantly improves the success rates of cannulation in difficult cases [4]. However, concerns regarding increased risks of adverse events including bleeding, perforation, and particularly PEP were raised in many earlier studies, and as a result, NKS is used as a rescue technique when all other options have failed [12, 13].

Although NKS has long been considered an independent procedure-related risk factor of PEP, confounders like a prolonged procedure time, repetitive unintentional pancreatic cannulation, and excessive manipulation causing ampullary edema might have exaggerated the incidence of PEP because almost all studies on NKS were conducted in the setting of difficult cannulation [14]. In addition, recent clinical data support our theory that needle-knife precut techniques improve cannulation rates without increasing adverse event rates when implemented early in challenging cases [15]. These previous results suggest that the efficacy and safety of NKS should be reevaluated and verified to determine whether this technique might be used as a standard cannulation modality.

In this preliminary prospective study, we evaluated the incidence rate of PEP after NKS performed as an initial procedure for biliary access in patients at increased risk of PEP. We also evaluated the success rate of biliary cannulation and other safety issues associated with NKS in patients at high risk of PEP.

## 2. Patients and Methods

### 2.1. Patients

In this prospective, single-arm study, we enrolled the patients at high risk of PEP who underwent primary NKS from June 2018 to November 2019. Patients were eligible if they met one or more of the following inclusion criteria: (1) the provision of written informed consent and an age between 18 and 90 years, (2) a naïve ampulla, (3) suspected to have biliary obstruction or biliary disease, (4) need for ERCP for the treatment of biliary obstruction, and (5) having at least one of the following risk factors for PEP [16]: suspected sphincter of Oddi dysfunction (SOD), an age between 18 and 50 years, female, normal common bile duct diameter (≤9 mm), normal serum bilirubin level, an obese status (body mass index ≥ 30), or a history of acute pancreatitis. Exclusion criteria were as follows. (1) an age of <18 years; (2) pregnancy; (3) mental retardation; (4) sensitivity to contrast agents; (5) a history of sphincterotomy or a pancreatobiliary procedure; (6) ampulla of Vater cancer; (7) separate bile duct and pancreatic duct openings; (8) a difficult ampulla approach due to abdominal surgery including stomach cancer with Billroth II anastomosis; (9) one or more of the following pancreatic diseases: acute pancreatitis within 30 days prior to enrollment, idiopathic acute recurrent pancreatitis, pancreas divisum, obstructive chronic pancreatitis, and pancreatic cancer; or (10) periampullary diverticulum type I or II.

Informed consent for ERCP using NKS was obtained from all patients enrolled in the study, and the study protocol was approved by the institutional review board of our institution (IUH-IRB 2017-12-01) prior to initiation of the study. All methods were carried out in accordance with relevant guidelines and regulations of our institution.

### 2.2. Endoscopic Procedure

All ERCP procedures were performed by either one of two endoscopists (S.J. and J.S.P) who had performed >300 ERCPs per year over the previous 5 years. ERCP was performed with a standard duodenoscope (TJF-260; Olympus Optical Co Ltd, Tokyo, Japan). Protease inhibitors and rectal indomethacin were not administered before or after ERCP because they might have affected the occurrence of post-ERCP pancreatitis. Before the procedure, midazolam and meperidine hydrochloride were administered intravenously for conscious sedation.

**Figure 1:**
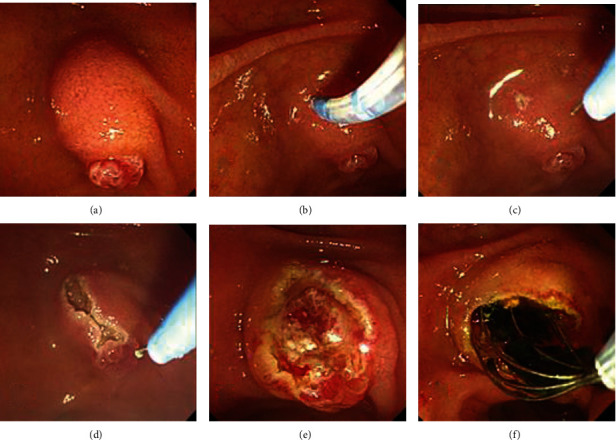
Needle-knife sphincterotomy procedure. (a) The major duodenal papilla is reached with a duodenoscope. (b) The needle-knife sphincterotome is introduced to perform an incision on the infundibulum. (c) The needle tip is advanced about 3-4 mm from the sheath and moved toward the bulging portion of the common bile duct. (d) The precut incision is formed from the top of infundibulum to the papilla orifice in a downward direction. (e) The pinkish mucosa of the bile duct is visualized after the incision. (f) The CBD stones are removed successfully using a basket without expanding the incision.

NKS procedures were performed using a needle-knife papillotome (MicroKnife XL; Boston Scientific, Natick, Mass) as follows. The needle tip was advanced about 1-2 mm from the sheath. For an obviously bulging papilla, a 1 to 2 mm-sized puncture was placed on top of the infundibulum using a needle knife. The cut was then slowly extended downward toward the 11 to 12 o'clock position along the ridge axis of the speculated intraduodenal portion of the CBD and stopped right before the papillary orifice. Cutting was continued gradually to a depth of about 2 to 3 mm from the mucosal surface and stopped when bile juice, pinkish mucosa of the bile duct, or whitish bulging of the sphincter of Oddi muscle was detected. If these were not observed during cutting, one or two additional incisions that deviated slightly from the presumed direction of the CBD were made (Video 1). Thereafter, a guidewire was inserted into the precut site for deep cannulation, and then, contrast dye (Ultravist, Iopromide, Bayer Schering Pharma, Berlin, Germany) was injected to confirm biliary cannulation. When the CBD was confirmed by contrast, the opening was expanded using a standard sphincterotome or dilation balloon catheter to a size determined by the endoscopist (Figure 1). Generator (VIO 300D, ERBE, Germany) settings for NKS were as follows: ENDO CUT I mode, effect 2, cut duration 3, cut interval 3, and 60 W.

### 2.3. Clinical Data after ERCP

Clinical data were collected prospectively during the first 3 days after ERCP procedures. Symptoms and vital signs were documented, and physical examinations were performed every 8 hours. Hematologic testing, which included serum amylase, liver enzymes, and complete blood cell counts, was performed before and 24 and 72 hours after the procedure to evaluate ERCP-related complications.

## 3. Measurement of Outcomes

### 3.1. Efficacy of NKS

Technical success was defined as successful biliary cannulation within 10 minutes since beginning of the incision using a needle-knife papillotome. Procedure time for biliary access was defined as time from incision start using the needle knife to CBD cannulation with the guidewire. Total procedure time was defined as time from duodenal intubation to procedure termination.

### 3.2. Safety of NKS

Primary outcome was defined as the incidence of PEP in the current study. Overall adverse events, that is, cholangitis, bleeding or perforation, or PEP, were assessed as secondary outcome. PEP was defined as the presence of abdominal pain with post-ERCP amylase elevation, which was defined as serum amylase elevation to more than three times normal and three times the pre-ERCP level. Abdominal CT images were acquired to identify PEP in patients with severe abdominal pain with an increased amylase or lipase level or with leukocytosis. Symptomatic hyperamylasemia was defined as at least a 3-fold increase in serum amylase without epigastric pain. Cholangitis was diagnosed based on a body temperature of >38°C for over 24 hours with abdominal pain. Perforation was defined as the presence of free air or contrast leakage as determined by a radiologic examination. Bleeding was defined as clinical evidence of bleeding, such as melena or hematemesis, with an associated decrease of 2 g/dL in hemoglobin concentration.

## 4. Results

### 4.1. Baseline Characteristics

Forty patients who had at least one of the patient-related risk factors for PEP were diagnosed as having biliary diseases which required ERCP. Six of the 40 were excluded due to large periampullary diverticulum (type I or II, *n* = 5) or separate bile duct and pancreatic duct openings (*n* = 1). Accordingly, 34 patients were enrolled in this prospective study. Patient baseline characteristics are shown in Table 1. Median patient age was 52 years (range, 25-88), and 24 patients (70.5%) were female. The most common reason for ERCP was CBD stones (*n* = 30). Numbers of risk factors were 1 in 9 (26.4%), 2 in 7 (20.6%), 3 in 8 (23.6%), 4 in 7 (20.6%), and 5 in 3 (8.8%).

### 4.2. The Efficacy of NKS

NKS was successfully performed in all 34 patients without any immediate adverse event. The technical success rate of biliary cannulation was 94.1% (32/34). Biliary cannulation by NKS was unsuccessful in 2 cases because distal ends of bile ducts, which usually manifest as a protuberant rubbery spot, were not detected even after making an incision in the infundibulum. The two patents involved were treated using a percutaneous approach. Pancreatic duct cannulation did not occur in all patients, and thus, pancreatic duct stent was not counted in the current study. Median procedure time for NKS was 4.1 minutes (range, 0.5-25.2). After the biliary cannulation, therapeutic endoscopic procedures, such as CBD stone retrieval with a balloon catheter and/or stone basket and biliary drainage with a plastic stent, were successfully performed in all patients. The median total procedure time was 11.3 minutes (range, 3.8–40.4, Table 2).

### 4.3. NKS-Related Adverse Events

No patient developed pancreatitis after ERCP using NKS. Abdominal pain was present in 3 of the 32 patients (9.4%) without increased pancreatic enzymes, and asymptomatic hyperamylasemia occurred in 1 patient (3.1%). All four recovered spontaneously without specific treatment. Bleeding was observed in 1 patient (3.1%) but did not affect hemoglobin concentration and was easily controlled by extraction balloon compression. Perforation, cholecystitis, and cholangitis did not occur (Table 3).

## 5. Discussion

The present study suggests primary NKS might be a reasonable procedure for biliary access in patients that require ERCP, especially in patients at increased risk of PEP. None of the patients in the current study developed PEP despite being at increased risk. Although post-ERCP adverse events such as abdominal pain, bleeding, or hyperamylasemia were observed in small number of patients, all patients recovered without any specific treatment. Moreover, NKS had a high success rate of biliary cannulation and facilitated ERCP to achieve the aims of endoscopic sessions, that is, biliary drainage, stone removal, and stent insertion.

NKS is a frequently used modality that has been reported to improve successful cannulation rates significantly, but it has also been associated with high ERCP-related morbidity, particularly an increased incidence of PEP [12, 13]. As a result, NKS is often reserved as a procedure of last resort. Several previous studies have suggested that this precut procedure is an independent risk factor of PEP [17]. However, the nature of this relationship and the mechanisms responsible are not completely understood. NKS is usually used only in selected cases of difficult biliary cannulation after all other standard cannulation techniques have failed; thus, preceding papillary trauma, edema, and inflammation after repeated attempts are common before the initiation of NKS and these factors are well-known procedure-related risk factors of PEP [18]. As a result, debate continues as to whether the increased risk of pancreatitis associated with NKS is actually due to precut itself or manipulation of the papilla before precut. In the present study, NKS was applied as initial biliary access technique and PEP did not develop in any patient, which suggests NKS *per se* is not a major etiologic factor of pancreatitis following ERCP. We consider that difficult cannulation, rather than NKS, of the sphincter plays a leading role in the development of PEP. When cannulation is difficult, it could lead to papillary edema, spasm, or even trauma and result in pancreatic duct obstruction and PEP. This suggestion is supported by a recent meta-analysis of seven randomized controlled studies [19], in which it was concluded the early application of NKS (3.9%) might reduce the incidence of PEP as compared with continued standard attempts (6.1%). Another meta-analysis also showed a significant reduction in the risk of PEP when the precut approach was implemented early during the procedure (odds ratio: 0.57, 95% confidential interval: 0.36-0.92, *P* = 0.02) [20]. However, concern was expressed regarding thermal injury of the pancreatic orifice during NKS, which can evoke pancreatitis. In the present study, the method of NKS was standardized in that the incision was started from the top of the infundibulum and downward to the ampulla orifice. The classic deroofing technique that involves cutting from the papillary orifice upward was not applied in the current study. Therefore, the pancreatic duct was not directly touched with the needle knife and the risk of direct thermal injury to the pancreatic duct was minimized. In addition, usually, NKS facilitates cannulation in the majority of cases considered difficult to cannulate. Consequently, we believe these factors act to prevent PEP after NKS and that NKS may be used as an initial procedure for biliary access in patients at increased risk of PEP.

Despite an acceptable success rate (94.1%) of NKS for biliary cannulation, NKS is sometimes technically challenging because it is basically a blind procedure [21]. The intraduodenal portion of CBD is not always located at its expected position in the infundibulum, and thus, cannulation failure may occur even after proper incision. In the present study, NKS failed in two patients; both had a slightly distorted ampulla. Although we tried to find an appropriate endoscopic view to begin the initial incision at a center of the protuberant portion of infundibulum and maintain the needle knife at a right angle during incision, we found it technically challenging to locate the knife at the proper position, and consequently, cutting the infundibulum diagonally failed to expose the bile duct. Therefore, if the ampulla is distorted, we believed that another cannulation technique, such as endoscopic ultrasound-guided intervention or the percutaneous approach is more suitable than NKS. In addition, we recommend NKS be performed by experienced hands because beginners find it difficult to decide between making a further incision or stopping the procedure which could lead to adverse event including perforation. Li et al. conducted study to determine the number of procedures required before effective and safe NKS can achieved [22]. In the results, at least 13 sessions of NKS were required to achieve a sustained success rate of biliary cannulation greater than 85%, and 50 sessions of NKS were required to reduce the incidence adverse event below 5%. Therefore, we recommend NKS to experienced endoscopist who performed a minimum of six precut procedures to achieve satisfactory outcomes.

NKS is a well-known independent risk factor of adverse events, such as cholangitis, bleeding, and perforation, after ERCP [23]. However, the risk of NKS-related adverse events as determined in the current study was satisfactory; cholangitis and perforation did not occur, and minor bleeding, which did not require transfusion or an endoscopic procedure, occurred in only one case. This low incidence of adverse events may have been due to a cautious incision technique as incisions for cannulation were made using a repetitive, fine cutting procedure and not using a single incision. In addition, choosing a knife with a 3 mm incision tip and maintaining an incision depth of 2-3 mm prevented perforation. If oozing bleeding was encountered during cutting procedure, the incision was stopped and resumed after hemostasis had been achieved by balloon compression or by using an epinephrine spray.

This study has several limitations. First, as a result of its small sample size, generalizations of its outcomes are limited; a large, prospective, randomized, multicenter study is required on this topic. Second, the study is limited by its single-center design and thus, potential operator-dependent bias. For example, the deroofing technique frequently used at other institutions was not performed in the current study. Thus, there is a risk that our results might not reflect the efficacy and safety of NKS in real clinical settings. Third, periampullary diverticulum types I and II were excluded, and thus, we did not explore relations between NKS outcomes and diverticulum type. Finally, SOD was not evaluated fully before enrollment. However, inserting manometry into the bile duct to confirm SOD is an invasive technique and might be a risk factor for PEP, and thus, intraductal pressure was not measured. Nevertheless, we assumed that SOD is probably associated with a low risk of PEP.

We conclude that NKS may be considered a feasible first modality for biliary access that can minimize the risk of PEP in patients deemed to be at high PEP risk if the procedure is performed by an experienced endoscopist. A large-scale, prospective, randomized, controlled study is warranted to verify the outcomes of this preliminary study.

## Figures and Tables

**Table 1:** Patient baseline characteristics.

Variable	Total (*n* = 34)
Age (year), median (range)	52 (25-88)
Sex, female, *n* (%)	24 (70.6)
*Causes of ERCP,n(%)*	
CBD stone	30 (88.2)
Malignant stricture	1 (2.9)
Benign biliary stricture	3 (8.8)
*Risk factors of PEP,n(%)*	
Young age (≤50 yrs)	14 (41.2)
Female	24 (70.5)
SOD	3 (8.8)
Normal CBD diameter (≤9 mm)	23 (67.6)
Normal total serum bilirubin	18 (52.9)
Previous acute pancreatitis	2 (5.9)
High BMI (>30 kg/m^2^)	4 (11.8)

ERCP: endoscopic retrograde cholangiopancreatography; CBD: common bile duct; PEP: post-ERCP pancreatitis; SOD: sphincter of Oddi dyskinesia; BMI: body mass index.

**Table 2:** Clinical outcomes of primary NKS.

Variable	Total (*n* = 34)
Technical success rate, *n* (%)	32 (94.1)
*The success rate of procedure after cannulation,n(%)*	
Stone removal	28 (100)
Biliary drainage	4 (100)
NKS procedure time (min), median (range)	4.1 (0.5-25.2)
Total procedure time (min), median (range)	11.3 (3.8–40.4)

ERCP: endoscopic retrograde cholangiopancreatography; NKS: needle-knife sphincterotomy.

**Table 3:** Adverse event post-ERCP.

Variable	Total (*n* = 32)
Pancreatitis	0
Bleeding	1 (3.1)
Asymptomatic hyperamylasemia	1 (3.1)
Cholangitis or cholecystitis	0
Perforation	0
Abdominal pain	3 (9.4)

ERCP: endoscopic retrograde cholangiopancreatography.

## Data Availability

The figure and table data used to support the findings of this study are included within the article.

## Supplementary Materials

Supplementary Materials Video clip: to show the process of needle-knife sphincterotomy for biliary access.
