# Supplementary figures and images for: Primary Needle-Knife Sphincterotomy for Biliary Access in Patients at High Risk of Post-Endoscopic Retrograde Cholangiopancreatography Pancreatitis
Source: Gastroenterol Res Pract. 2021 May 18;2021:6662000. doi: 10.1155/2021/6662000 (PMC8149254; doi:10.1155/2021/6662000)

## Slide 1
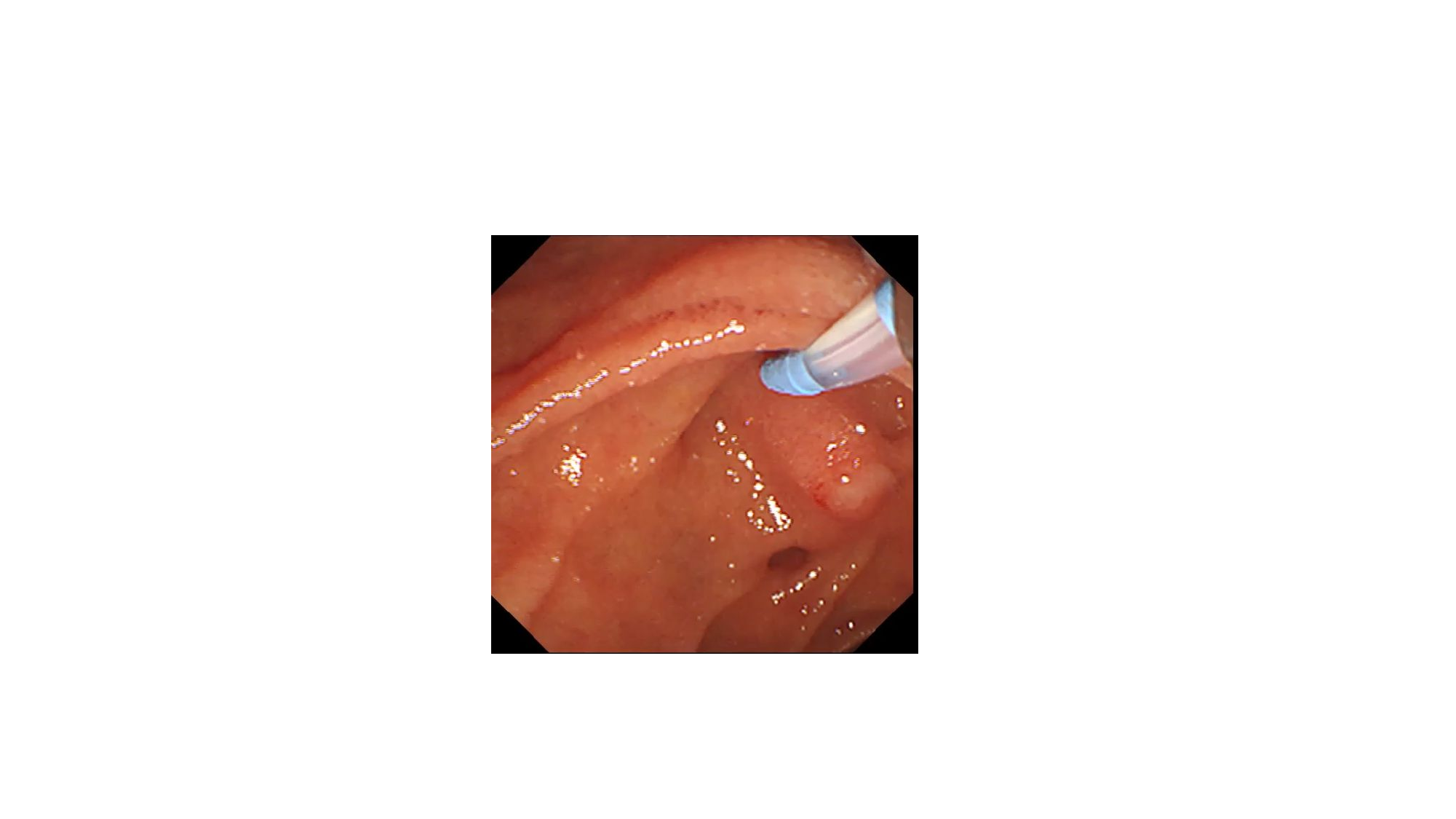

#

Supplement: Supplementary Materials — Video clip: to show the process of needle-knife sphincterotomy for biliary access. [file 6662000.f1.pptx]
